# Supplementary material for: Economic Impact of a Precision Nutrition Digital Therapeutic on Employer Health Costs: A Multi-Employer and Multi-Year Claims Analysis
Source: Healthcare (Basel). 2025 Dec 2;13(23):3147. doi: 10.3390/healthcare13233147 (PMC12691840; doi:10.3390/healthcare13233147)
Supplement: Supplementary file 1 [file healthcare-13-03147-s001.zip › Figure S1.pdf]

Supp Figure S1. Flowchart of claims processing, sample selection, panel construction and two-stage difference-in-differences estimation (DiD2S). The flowchart shows (A) raw claims ingestion and exclusion rules, (B) de-identification and QC, (C) treated assignment and control selection by employer × calendar-month strata, (D) strata retention rules used to ensure contemporaneous treated and control observations, (E) aggregation to the member–month panel and the Stage-1/Stage-2 DiD pipeline, and (F) robustness checks and output tables/figures. Controls were selected by employer × calendar-month strata. See Methods 2.5–2.6 and Supplementary Tables for full details.

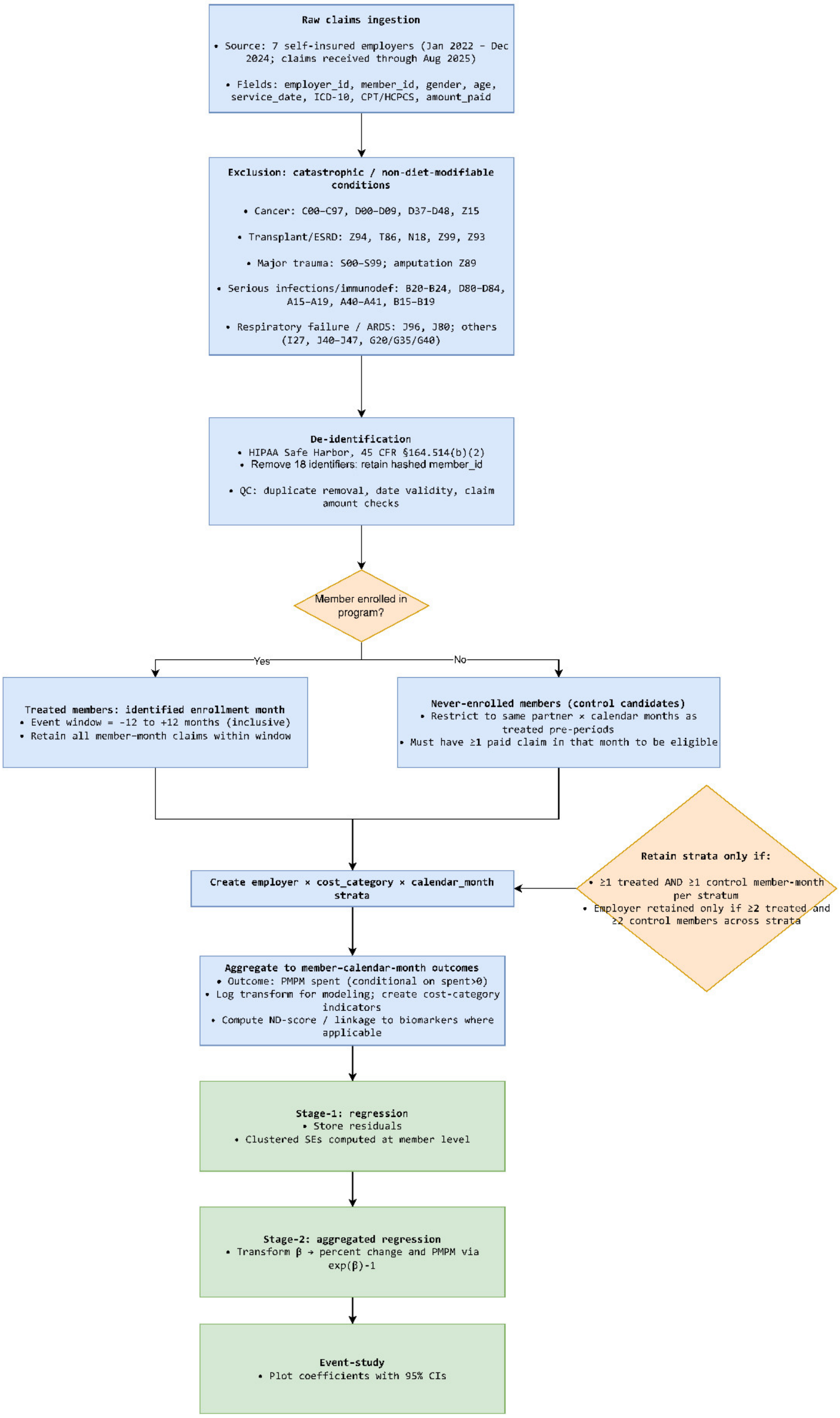
Raw claims ingestion
• Source: 7 self-insured employers (Jan 2022 – Dec 2024; claims received through Aug 2025)
• Fields: employer_id, member_id, gender, age, service_date, ICD-10, CPT/HCPCS, amount_paid
Exclusion: catastrophic / non-diet-modifiable conditions
• Cancer: C00–C97, D00–D09, D37–D48, Z15
• Transplant/ESRD: Z94, T86, N18, Z99, Z93
• Major trauma: S00–S99; amputation Z89
• Serious infections/immunodef: B20–B24, D80–D84, A15–A19, A40–A41, B15–B19
• Respiratory failure / ARDS: J96, J80; others (I27, J40–J47, G20/G35/G40)
De-identification
• HIPAA Safe Harbor, 45 CFR §164.514(b)(2)
• Remove 18 identifiers: retain hashed member_id
• QC: duplicate removal, date validity, claim amount checks
Member enrolled in program?
Yes
No
Treated members: identified enrollment month
• Event window = -12 to +12 months (inclusive)
• Retain all member-month claims within window
Never-enrolled members (control candidates)
• Restrict to same partner × calendar months as treated pre-periods
• Must have ≥1 paid claim in that month to be eligible
Retain strata only if:
• ≥1 treated AND ≥1 control member-month per stratum
• Employer retained only if ≥2 treated and ≥2 control members across strata
Create employer × cost_category × calendar_month strata
Aggregate to member-calendar-month outcomes
• Outcome: PMPM spent (conditional on spent>0)
• Log transform for modeling; create cost-category indicators
• Compute ND-score / linkage to biomarkers where applicable
Stage-1: regression
• Store residuals
• Clustered SEs computed at member level
Stage-2: aggregated regression
• Transform β → percent change and PMPM via exp(β)-1
Event-study
• Plot coefficients with 95% CIs
