# Supplementary material for: Economic Impact of a Precision Nutrition Digital Therapeutic on Employer Health Costs: A Multi-Employer and Multi-Year Claims Analysis
Source: Healthcare (Basel). 2025 Dec 2;13(23):3147. doi: 10.3390/healthcare13233147 (PMC12691840; doi:10.3390/healthcare13233147)
Supplement: Supplementary file 1 [file healthcare-13-03147-s001.zip › Table S1.pdf]

Supplementary Table S1. Association of program enrollment with healthcare spending by CPT/HCPCS family, stratified by disease domain.

Estimates are presented on the log scale with 95% confidence intervals (CI), alongside the corresponding percentage change and absolute per-member-per-month (PMPM) change in costs. Negative values represent reductions in spending. The table reports results only for CPT families with sufficient sample size and relevance in each disease category. *p-values* are based on regression models with treatment-control comparisons in the 12-month pre/post window. "Enrolled (N)" refers to the number of treated members with at least one claim in the category, "Never-Enrolled (N)" refers to matched controls, and "Employers (N)" reflects the number of participating employer groups contributing data for that cell.

**Cost Category**	**CPT/HCPCS Codes Family**	**Estimate (95% CI, log scale)**	**% Change (95% CI)**	**PMPM Change (95% CI)**	**p-value**	**Enrolled (N)**	**Never-Enrolled (N)**	**Employers (N)**
Obesity								
	Office EM	-0.127 (-0.456, 0.202)	-12 (-37, 22)	-17 (-53, 32)	0.449	13	568	3
Digestive Health								
	Medical nutrition therapy	-2.653 (-3.786, -1.519)	-93 (-98, -78)	-283 (-297, -238)	5.41E-06	8	158	1
Mental Health								
	Psychotherapy individual	-0.054 (-0.266, 0.158)	-5 (-23, 17)	-15 (-68, 50)	0.617	29	708	3
	Psychotherapy addon with EM	-0.206 (-0.358, -0.055)	-19 (-30, -5)	-21 (-34, -6)	0.008	8	149	1
Anxiety								
	Psychotherapy individual	0.293 (-0.128, 0.714)	34 (-12, 104)	91 (-32, 279)	0.173	9	216	2
	Psychotherapy addon with EM	-0.172 (-0.269, -0.076)	-16 (-24, -7)	-18 (-27, -8)	5.18E-04	6	107	1
Depression								
	Psychotherapy individual	-0.305 (-0.965, 0.354)	-26 (-62, 42)	-77 (-182, 125)	0.364	6	129	2
	Psychotherapy addon with EM	-0.226 (-0.423, -0.029)	-20 (-34, -3)	-23 (-39, -3)	0.025	6	77	1
